# (Dimethyl sulfoxide-κ*O*)trimeth­yl(2-methyl-3,5-dinitro­benzoato-κ*O* ^1^)tin(IV)

**DOI:** 10.1107/S1600536811022240

**Published:** 2011-06-18

**Authors:** Muhammed Danish, Sabiha Ghafoor, Nazir Ahmad, Wojciech Starosta, Janusz Leciejewicz

**Affiliations:** a Department of Chemistry, University of Gujrat, Hafiz Hayat Campus, Gujrat 50700, Pakistan; b Department of Chemistry, University of Sargodha, Sargodha 40100, Pakistan; c Institute of Nuclear Chemistry and Technology, ul. Dorodna 16, 03-195 Warszawa, Poland

## Abstract

In the title mononuclear complex, [Sn(CH_3_)_3_(C_7_H_5_N_2_O_6_)(C_2_H_6_OS)], the Sn^IV^ ion is coordinated by three methyl groups in the equatorial plane, and by an O atom from a 2-methyl-3,5-dinitrobenzoate ligand and a dimethyl sulfoxide ligand in the axial sites, to form a slightly distorted trigonal–bipyramidal environment. The O atoms of one of the nitro groups are disordered over two sets of sites, with refined occupancies of 0.55 (4) and 0.45 (4). The closest inter­molecular inter­action is a weak C—H⋯O hydrogen bond.

## Related literature

For the applications of trimethytin complexes, see: Gielen *et al.* (2005 ▶); Gielen (2002 ▶); Hameed *et al.* (2009 ▶); Ashhad *et al.* (2005 ▶). For the structure of a trimethyl­tin complex with a 2-methyl­benzene-3-carboxyl­ate ligand, see: Danish *et al.* (2010 ▶). For the structure of a triphenyl­tin complex with 2-methyl-3,5-dinitro­benzene carboxyl­ate and methanol ligands, see: Danish *et al.* (2011 ▶).
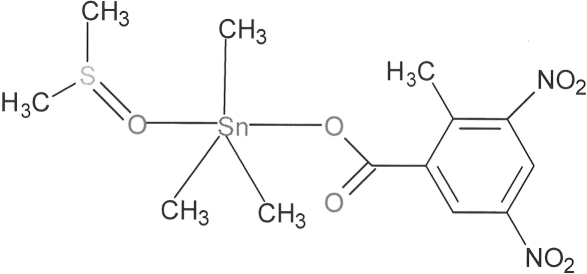


## Experimental

### 

#### Crystal data

[Sn(CH_3_)_3_(C_7_H_5_N_2_O_6_)(C_2_H_6_OS)]
*M* *_r_* = 467.06
Monoclinic, 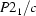

*a* = 9.6180 (19) Å
*b* = 12.971 (3) Å
*c* = 15.612 (3) Å
β = 102.98 (3)°
*V* = 1897.9 (7) Å^3^
*Z* = 4
Mo *K*α radiation
μ = 1.49 mm^−1^
*T* = 293 K
0.42 × 0.24 × 0.08 mm

#### Data collection

Kuma KM-4 four-circle diffractometer
Absorption correction: analytical (*CrysAlis RED*; Oxford Diffraction, 2008 ▶) *T*_min_ = 0.753, *T*_max_ = 0.890
4755 measured reflections
4500 independent reflections
2464 reflections with *I* > 2σ(*I*)
*R*_int_ = 0.030
3 standard reflections every 200 reflections  intensity decay: 0.2%

#### Refinement

*R*[*F* ^2^ > 2σ(*F* ^2^)] = 0.046
*wR*(*F* ^2^) = 0.144
*S* = 1.01
4500 reflections
242 parameters
4 restraints
H-atom parameters constrained
Δρ_max_ = 1.55 e Å^−3^
Δρ_min_ = −1.86 e Å^−3^

### 

Data collection: *KM-4 Software* (Kuma, 1996 ▶); cell refinement: *KM-4 Software*; data reduction: *DATAPROC* (Kuma, 2001 ▶); program(s) used to solve structure: *SHELXS97* (Sheldrick, 2008 ▶); program(s) used to refine structure: *SHELXL97* (Sheldrick, 2008 ▶); molecular graphics: *SHELXTL* (Sheldrick, 2008 ▶); software used to prepare material for publication: *SHELXTL*.

## Supplementary Material

Crystal structure: contains datablock(s) I, global. DOI: 10.1107/S1600536811022240/lh5258sup1.cif

Structure factors: contains datablock(s) I. DOI: 10.1107/S1600536811022240/lh5258Isup2.hkl

Additional supplementary materials:  crystallographic information; 3D view; checkCIF report

## Figures and Tables

**Table 1:** Hydrogen-bond geometry (Å, °)

*D*—H⋯*A*	*D*—H	H⋯*A*	*D*⋯*A*	*D*—H⋯*A*
C11—H11*C*⋯O3^i^	0.96	2.59	3.509 (15)	162

Symmetry code: (i) 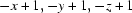.

**Fig. 1.**
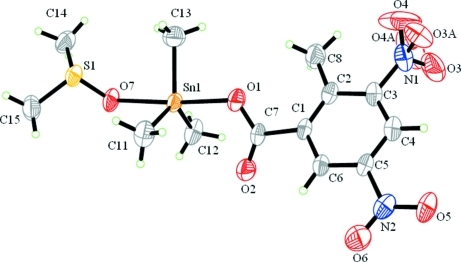
The molecular structure with 50% probability displacement ellipsoids. Atoms labeled O3A and O4A are the minor components of disorder.

## supplementary crystallographic information

### Comment

Organotin carboxylates are extensively studied bexcause of their potential diversified biological applications. Trimethyltin carboxylates find use as antifungal (Gielen *et al.*, 2005; Gielen, 2002) and antibacterial agents (Hameed *et al.*, 2009; Ashhad *et al.*, 2005). The title compound is part of our continued effort in this area (Danish *et al.*, 2010, 2011). The structure of the title compound is composed of mononuclear molecules in which an Sn atom is coordinated by three methyl C atoms, one carboxylato O atom donated by a 2-methyl-3,5-dinitrobenzenecarboxylate ligand and an O atom from a DMSO ligand forming slightly distorted trigonal bipyramidal environment. The methyl C atoms form the equatorial plane. The Sn atom is displaced by 0.1082 (2) Å from the plane towards the carboxylate O atom. The latter and the DMSO O atom are in the axial sites. The Sn—C bond lengths range from 2.097 (3) to 2.121 (3) Å and are close to those reported earlier in the structures of other trimethyltin complexes (*e.g.* Danish *et al.*, 2010). The 2-methyl-3,5-dintro-benzenecarboxylate ligand molecule is essentially planar with an r.m.s. deviation of 0.0041 (1) Å. The O atoms of one of the nitro groups show positional disorder with a major component occupancy of 0.55 (4). The other nitro group forms a dihedral angle of 5.9 (2)° with the methylbenzene ring. The carboxylate group donates a single O atom to the Sn atom. The observed Sn—O bond length of 2.106 (3) Å is typical (*e.g.* Danish *et al.*, 2010, 2011). The closest intermolecular interaction is a weak C—H···O hydrogen bond.

### Experimental

The sodium salt of 3,5-dinitro-*o*-toluic acid (2.48 g, 0.01 mol) was suspended in 25 ml of dry chloroform contained in a 100 ml round-bottom flask; trimethyltin chloride (2.00 g, 0.01 mol) was dissolved in 25 ml of dry chloroform was then added dropwise with constant stirring at room temperature. The reaction mixture was then refluxed in chloroform for 6 h and then brought to room temperature. Filtration was carried out to remove sodium chloride formed during the reaction. After evaporation, the solid mass was recrystallized from DMSO. m.p. 395 K; yield 88%.

### Refinement

H atoms were placed in calculated positions with C—H = 0.93 for the benzene H atoms and 0.96 Å for methyl groups and treated as riding on the parent atoms with *U*_iso_(H) = 1.2*U*_eq_(C) or 1.5*U*_eq_(C_methyl_).

### Crystal data

**Table:**

[Sn(CH_3_)_3_(C_7_H_5_N_2_O_6_)(C_2_H_6_OS)]	*F*(000) = 936
*M**_r_* = 467.06	*D*_x_ = 1.635 Mg m^−^^3^
Monoclinic, *P*2_1_/*c*	Mo *K*α radiation, λ = 0.71073 Å
Hall symbol: -P 2ybc	Cell parameters from 25 reflections
*a* = 9.6180 (19) Å	θ = 6–15°
*b* = 12.971 (3) Å	µ = 1.49 mm^−^^1^
*c* = 15.612 (3) Å	*T* = 293 K
β = 102.98 (3)°	Plate, pale yellow
*V* = 1897.9 (7) Å^3^	0.42 × 0.24 × 0.08 mm
*Z* = 4	

### Data collection

**Table:**

Kuma KM-4 four-circle diffractometer	2464 reflections with *I* > 2σ(*I*)
Radiation source: fine-focus sealed tube	*R*_int_ = 0.030
graphite	θ_max_ = 28.0°, θ_min_ = 2.1°
Profile data from ω/2θ scans	*h* = 0→12
Absorption correction: analytical (*CrysAlis RED*; Oxford Diffraction, 2008)	*k* = 0→17
*T*_min_ = 0.753, *T*_max_ = 0.890	*l* = −19→19
4755 measured reflections	3 standard reflections every 200 reflections
4500 independent reflections	intensity decay: 0.2%

### Refinement

**Table:**

Refinement on *F*^2^	Primary atom site location: structure-invariant direct methods
Least-squares matrix: full	Secondary atom site location: difference Fourier map
*R*[*F*^2^ > 2σ(*F*^2^)] = 0.046	Hydrogen site location: inferred from neighbouring sites
*wR*(*F*^2^) = 0.144	H-atom parameters constrained
*S* = 1.01	*w* = 1/[σ^2^(*F*_o_^2^) + (0.0947*P*)^2^] where *P* = (*F*_o_^2^ + 2*F*_c_^2^)/3
4500 reflections	(Δ/σ)_max_ = 0.001
242 parameters	Δρ_max_ = 1.55 e Å^−^^3^
4 restraints	Δρ_min_ = −1.86 e Å^−^^3^

### Special details

**Table:**

Geometry. All e.s.d.'s (except the e.s.d. in the dihedral angle between two l.s. planes) are estimated using the full covariance matrix. The cell e.s.d.'s are taken into account individually in the estimation of e.s.d.'s in distances, angles and torsion angles; correlations between e.s.d.'s in cell parameters are only used when they are defined by crystal symmetry. An approximate (isotropic) treatment of cell e.s.d.'s is used for estimating e.s.d.'s involving l.s. planes.
Refinement. Refinement of *F*^2^ against ALL reflections. The weighted *R*-factor *wR* and goodness of fit *S* are based on *F*^2^, conventional *R*-factors *R* are based on *F*, with *F* set to zero for negative *F*^2^. The threshold expression of *F*^2^ > σ(*F*^2^) is used only for calculating *R*-factors(gt) *etc*. and is not relevant to the choice of reflections for refinement. *R*-factors based on *F*^2^ are statistically about twice as large as those based on *F*, and *R*-factors based on ALL data will be even larger.

### Fractional atomic coordinates and isotropic or equivalent isotropic displacement parameters (Å^2^)

**Table:**

	*x*	*y*	*z*	*U*_iso_*/*U*_eq_	Occ. (<1)
Sn1	0.78174 (3)	0.49914 (2)	0.21581 (2)	0.04452 (13)	
S1	1.10224 (14)	0.53273 (12)	0.15598 (9)	0.0519 (3)	
O1	0.6418 (4)	0.4762 (3)	0.3068 (3)	0.0605 (10)	
C4	0.2430 (5)	0.4664 (4)	0.4708 (3)	0.0520 (11)	
H4	0.1832	0.4573	0.5094	0.062*	
C2	0.4266 (5)	0.3988 (4)	0.3974 (3)	0.0468 (11)	
C5	0.2474 (5)	0.5571 (4)	0.4271 (3)	0.0464 (11)	
C1	0.4264 (4)	0.4929 (3)	0.3564 (3)	0.0433 (9)	
O5	0.0655 (5)	0.6244 (4)	0.4846 (3)	0.0929 (15)	
N2	0.1513 (5)	0.6424 (4)	0.4406 (3)	0.0634 (11)	
O2	0.4719 (4)	0.5782 (3)	0.2313 (3)	0.0696 (11)	
C3	0.3313 (5)	0.3894 (4)	0.4546 (3)	0.0507 (11)	
C6	0.3363 (5)	0.5725 (4)	0.3706 (3)	0.0460 (10)	
H6	0.3364	0.6353	0.3420	0.055*	
C8	0.5152 (7)	0.3080 (4)	0.3837 (4)	0.0719 (16)	
H8A	0.4854	0.2844	0.3241	0.108*	
H8B	0.5032	0.2535	0.4230	0.108*	
H8C	0.6138	0.3278	0.3951	0.108*	
O7	0.9437 (4)	0.5132 (3)	0.1204 (3)	0.0582 (9)	
C11	0.8247 (6)	0.6531 (4)	0.2547 (4)	0.0705 (16)	
H11A	0.7725	0.6982	0.2102	0.106*	
H11B	0.9251	0.6662	0.2628	0.106*	
H11C	0.7963	0.6651	0.3090	0.106*	
C14	1.1886 (6)	0.4276 (5)	0.1203 (5)	0.080 (2)	
H14A	1.2873	0.4442	0.1249	0.121*	
H14B	1.1443	0.4123	0.0602	0.121*	
H14C	1.1816	0.3686	0.1562	0.121*	
C13	0.9183 (7)	0.3843 (5)	0.2860 (4)	0.0768 (18)	
H13A	0.8622	0.3330	0.3070	0.115*	
H13B	0.9831	0.4154	0.3350	0.115*	
H13C	0.9714	0.3527	0.2478	0.115*	
C15	1.1490 (7)	0.6276 (5)	0.0867 (5)	0.0784 (18)	
H15A	1.1406	0.5995	0.0289	0.118*	
H15B	1.2456	0.6492	0.1098	0.118*	
H15C	1.0862	0.6857	0.0838	0.118*	
C12	0.6354 (6)	0.4555 (6)	0.1006 (4)	0.0708 (15)	
H12A	0.6211	0.5118	0.0597	0.106*	
H12B	0.5461	0.4376	0.1146	0.106*	
H12C	0.6719	0.3971	0.0749	0.106*	
O6	0.1644 (5)	0.7246 (4)	0.4073 (4)	0.0974 (16)	
C7	0.5190 (5)	0.5193 (4)	0.2909 (4)	0.0508 (12)	
N1	0.3216 (6)	0.2921 (4)	0.5005 (3)	0.0681 (13)	
O4A	0.2078 (17)	0.2483 (18)	0.4785 (18)	0.104 (8)	0.45 (4)
O3A	0.414 (3)	0.258 (2)	0.555 (3)	0.175 (15)	0.45 (4)
O4	0.287 (4)	0.2135 (11)	0.4607 (9)	0.128 (9)	0.55 (4)
O3	0.3585 (19)	0.2947 (11)	0.5794 (5)	0.084 (5)	0.55 (4)

### Atomic displacement parameters (Å^2^)

**Table:**

	*U*^11^	*U*^22^	*U*^33^	*U*^12^	*U*^13^	*U*^23^
Sn1	0.03448 (18)	0.0566 (2)	0.0461 (2)	0.00010 (14)	0.01663 (12)	0.00159 (15)
S1	0.0403 (6)	0.0702 (7)	0.0495 (7)	−0.0029 (6)	0.0192 (5)	−0.0052 (6)
O1	0.048 (2)	0.077 (2)	0.065 (2)	0.0068 (17)	0.0314 (17)	0.0058 (18)
C4	0.046 (3)	0.063 (3)	0.051 (3)	−0.011 (2)	0.020 (2)	−0.007 (2)
C2	0.035 (2)	0.054 (3)	0.053 (3)	−0.009 (2)	0.0140 (19)	−0.011 (2)
C5	0.031 (2)	0.057 (3)	0.054 (3)	−0.004 (2)	0.0159 (19)	−0.005 (2)
C1	0.0305 (19)	0.059 (3)	0.043 (2)	−0.0075 (19)	0.0135 (16)	−0.004 (2)
O5	0.076 (3)	0.111 (4)	0.114 (4)	0.020 (3)	0.067 (3)	0.008 (3)
N2	0.051 (3)	0.075 (3)	0.069 (3)	0.008 (2)	0.024 (2)	−0.008 (2)
O2	0.056 (2)	0.093 (3)	0.067 (3)	0.011 (2)	0.0278 (19)	0.022 (2)
C3	0.044 (3)	0.058 (3)	0.054 (3)	−0.014 (2)	0.019 (2)	−0.004 (2)
C6	0.039 (2)	0.056 (3)	0.046 (3)	−0.004 (2)	0.018 (2)	0.000 (2)
C8	0.068 (4)	0.062 (3)	0.096 (4)	0.001 (3)	0.040 (3)	−0.002 (3)
O7	0.0427 (18)	0.085 (3)	0.053 (2)	−0.0081 (16)	0.0236 (15)	−0.0030 (17)
C11	0.063 (4)	0.070 (4)	0.085 (4)	−0.011 (3)	0.029 (3)	−0.014 (3)
C14	0.049 (3)	0.074 (4)	0.123 (6)	0.010 (3)	0.029 (4)	0.003 (4)
C13	0.070 (4)	0.094 (4)	0.072 (4)	0.023 (3)	0.030 (3)	0.023 (3)
C15	0.064 (4)	0.069 (4)	0.111 (5)	−0.011 (3)	0.039 (3)	0.003 (4)
C12	0.048 (3)	0.105 (4)	0.062 (3)	−0.014 (3)	0.019 (3)	−0.018 (3)
O6	0.096 (4)	0.070 (3)	0.144 (5)	0.024 (3)	0.065 (3)	0.014 (3)
C7	0.041 (2)	0.063 (3)	0.056 (3)	−0.005 (2)	0.026 (2)	−0.001 (2)
N1	0.074 (4)	0.065 (3)	0.074 (4)	−0.020 (3)	0.035 (3)	−0.001 (3)
O4A	0.104 (11)	0.084 (11)	0.126 (15)	−0.031 (9)	0.030 (9)	0.033 (9)
O3A	0.099 (15)	0.084 (15)	0.29 (3)	−0.014 (10)	−0.067 (17)	0.097 (17)
O4	0.22 (2)	0.076 (7)	0.109 (8)	−0.068 (10)	0.075 (11)	−0.036 (6)
O3	0.116 (10)	0.063 (7)	0.073 (8)	−0.003 (6)	0.018 (6)	0.019 (4)

### Geometric parameters (Å, °)

**Table:**

Sn1—C12	2.097 (5)	C8—H8B	0.9600
Sn1—C11	2.101 (5)	C8—H8C	0.9600
Sn1—C13	2.121 (6)	C11—H11A	0.9600
Sn1—O1	2.186 (4)	C11—H11B	0.9600
Sn1—O7	2.391 (4)	C11—H11C	0.9600
S1—O7	1.523 (4)	C14—H14A	0.9600
S1—C14	1.752 (6)	C14—H14B	0.9600
S1—C15	1.761 (6)	C14—H14C	0.9600
O1—C7	1.280 (6)	C13—H13A	0.9600
C4—C5	1.366 (7)	C13—H13B	0.9600
C4—C3	1.371 (7)	C13—H13C	0.9600
C4—H4	0.9300	C15—H15A	0.9600
C2—C1	1.379 (6)	C15—H15B	0.9600
C2—C3	1.420 (7)	C15—H15C	0.9600
C2—C8	1.497 (7)	C12—H12A	0.9600
C5—C6	1.374 (6)	C12—H12B	0.9600
C5—N2	1.486 (6)	C12—H12C	0.9600
C1—C6	1.398 (6)	N1—O3A	1.174 (9)
C1—C7	1.537 (6)	N1—O3	1.205 (8)
O5—N2	1.210 (6)	N1—O4	1.201 (8)
N2—O6	1.205 (6)	N1—O4A	1.213 (8)
O2—C7	1.210 (6)	O4A—O4	0.981 (17)
C3—N1	1.464 (7)	O3A—O3	0.87 (4)
C6—H6	0.9300	O3A—O4	1.78 (2)
C8—H8A	0.9600		
			
C12—Sn1—C11	123.7 (3)	H11B—C11—H11C	109.5
C12—Sn1—C13	118.2 (3)	S1—C14—H14A	109.5
C11—Sn1—C13	117.3 (3)	S1—C14—H14B	109.5
C12—Sn1—O1	97.35 (19)	H14A—C14—H14B	109.5
C11—Sn1—O1	92.95 (19)	S1—C14—H14C	109.5
C13—Sn1—O1	88.19 (19)	H14A—C14—H14C	109.5
C12—Sn1—O7	83.81 (18)	H14B—C14—H14C	109.5
C11—Sn1—O7	89.68 (18)	Sn1—C13—H13A	109.5
C13—Sn1—O7	87.77 (19)	Sn1—C13—H13B	109.5
O1—Sn1—O7	175.87 (12)	H13A—C13—H13B	109.5
O7—S1—C14	105.2 (3)	Sn1—C13—H13C	109.5
O7—S1—C15	105.4 (3)	H13A—C13—H13C	109.5
C14—S1—C15	98.3 (3)	H13B—C13—H13C	109.5
C7—O1—Sn1	119.4 (3)	S1—C15—H15A	109.5
C5—C4—C3	116.5 (5)	S1—C15—H15B	109.5
C5—C4—H4	121.7	H15A—C15—H15B	109.5
C3—C4—H4	121.7	S1—C15—H15C	109.5
C1—C2—C3	115.8 (4)	H15A—C15—H15C	109.5
C1—C2—C8	124.8 (4)	H15B—C15—H15C	109.5
C3—C2—C8	119.4 (5)	Sn1—C12—H12A	109.5
C4—C5—C6	122.6 (5)	Sn1—C12—H12B	109.5
C4—C5—N2	118.8 (4)	H12A—C12—H12B	109.5
C6—C5—N2	118.7 (4)	Sn1—C12—H12C	109.5
C2—C1—C6	121.1 (4)	H12A—C12—H12C	109.5
C2—C1—C7	124.5 (4)	H12B—C12—H12C	109.5
C6—C1—C7	114.4 (4)	O2—C7—O1	126.5 (5)
O6—N2—O5	124.6 (5)	O2—C7—C1	118.6 (4)
O6—N2—C5	117.9 (4)	O1—C7—C1	114.9 (5)
O5—N2—C5	117.5 (5)	O3A—N1—O3	43 (2)
C4—C3—C2	124.5 (5)	O3A—N1—O4	97.4 (14)
C4—C3—N1	115.3 (5)	O3—N1—O4	122.4 (10)
C2—C3—N1	120.2 (5)	O3A—N1—O4A	121.3 (13)
C5—C6—C1	119.5 (4)	O3—N1—O4A	110.0 (13)
C5—C6—H6	120.2	O4—N1—O4A	48.0 (8)
C1—C6—H6	120.2	O3A—N1—C3	124.0 (11)
C2—C8—H8A	109.5	O3—N1—C3	116.1 (7)
C2—C8—H8B	109.5	O4—N1—C3	121.3 (8)
H8A—C8—H8B	109.5	O4A—N1—C3	114.8 (8)
C2—C8—H8C	109.5	O4—O4A—N1	65.4 (8)
H8A—C8—H8C	109.5	O3—O3A—N1	70.4 (12)
H8B—C8—H8C	109.5	O3—O3A—O4	99.4 (19)
S1—O7—Sn1	121.6 (2)	N1—O3A—O4	41.9 (8)
Sn1—C11—H11A	109.5	O4A—O4—N1	66.6 (8)
Sn1—C11—H11B	109.5	O4A—O4—O3A	92.9 (14)
H11A—C11—H11B	109.5	N1—O4—O3A	40.7 (7)
Sn1—C11—H11C	109.5	O3A—O3—N1	66.6 (14)
H11A—C11—H11C	109.5		

### Hydrogen-bond geometry (Å, °)

**Table:**

*D*—H···*A*	*D*—H	H···*A*	*D*···*A*	*D*—H···*A*
C11—H11C···O3^i^	0.96	2.59	3.509 (15)	162.

Symmetry codes: (i) −*x*+1, −*y*+1, −*z*+1.
